# Post-viral Immune-Mediated Vasculitis Following Concurrent Dengue and Chikungunya Infection: A Case Report

**DOI:** 10.7759/cureus.83677

**Published:** 2025-05-07

**Authors:** Yaqoot Khan, Thanda Aung, Eric Liu

**Affiliations:** 1 Rheumatology, University of California Los Angeles David Geffen School of Medicine, Los Angeles, USA

**Keywords:** autoimmunity, chikungunya, dengue, foot drop, infection, mononeuritis multiplex, positive ana, systemic, vasculitis, virus

## Abstract

We present a case of a 78-year-old female who developed persistent neurological complications following a concurrent dengue and chikungunya viral infection. The patient experienced progressive weakness and ambulatory difficulties, culminating in left foot drop. This case highlights the potential long-term neurological sequelae of these tropical viral infections and emphasizes the importance of considering a post-viral vasculitis-like presentation in patients with delayed neurological symptoms.

## Introduction

Dengue and chikungunya infections are mosquito-borne viral diseases that have become increasingly prevalent in tropical and subtropical regions worldwide [1,2]. Both are transmitted primarily by Aedes mosquitoes and typically present with acute febrile illness [2,3]. While most cases resolve without long-term complications, neurological sequelae have been reported in the literature, including encephalitis, meningitis, Guillain-Barré syndrome, myelitis, and mononeuritis multiplex for dengue, and encephalopathy, myelopathy, neuropathy, and myopathy for chikungunya [4].

Co-infections with both viruses have been increasingly reported in endemic regions, posing significant diagnostic and management challenges [5]. The pathophysiology of the neurological manifestations is thought to involve both direct viral invasion of neural tissues and post-infectious immune-mediated mechanisms [6]. Post-viral vasculitis following these infections represents a particularly challenging diagnostic entity, with limited case reports in the literature.

This case presents an unusual manifestation of persistent neurological complications following concurrent infection with both viruses, highlighting the importance of recognizing delayed neurological sequelae and considering post-viral immune mechanisms in patients with the appropriate epidemiological and clinical context. This case has not been presented in any other abstracts, posters, or conferences.

## Case presentation

A 78-year-old female with a medical history of well-controlled diabetes, vitiligo of 40+ years duration, and hypertension presented to the emergency department with an eight-week history of progressive weakness and difficulty ambulating. Four months prior to presentation, while visiting Mexico from California, she experienced a febrile illness with nausea and weakness and was clinically diagnosed with both dengue and chikungunya infection. Following this episode, she developed a rash on her leg, with biopsy findings consistent with lymphocytic vasculitis. The biopsy was performed in Mexico and the slides were not available for review.

The patient reported unintentional weight loss of 20 pounds accompanied by poor appetite and progressive weakness that significantly impaired her ability to walk. Her review of systems was otherwise negative for Raynaud's phenomenon or dysphagia. She denied any gastrointestinal or urinary symptoms. 

Vital signs were stable. Physical examination revealed normal muscle bulk, no pain on palpation, reduced left foot dorsiflexion and quadriceps muscle strength (2/5), and the remainder of the examination was unremarkable. Her neurological examination revealed normal reflexes bilaterally, and a normal sensory exam. 

During her hospitalization, she was evaluated by neurology, rheumatology and infectious disease teams. She underwent extensive workups including laboratory tests, imaging and neurological testing. The initial hematology and chemistry panel was normal with a mildly elevated erythrocyte sedimentation rate (Table 1).

**Table 1:** Laboratory Investigations: Hematology and Chemistry Panel CPK, Creatine Phosphokinase; AST, Aspartate Transaminase; ALT, Alanine Transaminase; CRP, C-reactive Protein; RPR with TP-PA, Rapid Plasma Reagin Test with Treponema Pallidum confirmation; ESR, erythrocyte sedimentation rate.

Test	Observed Value	Reference Range
White Cell count	9.51	4.16-9.95 x10E3/uL
Hemoglobin	13.7	11.6-15.2 g/dL
Hematocrit	41.7	39.2-45.4%
Platelet count	209	143-398 x10E3/uL
Creatinine	0.59	0.60-1.3 mg/dl
CPK	40	38-282 U/L
Aldolase	3.5	1.2-7.6 U/L
AST	14	13-62 U/L
ESR	38	<25 mm/Hr
CRP	<0.3	<0.3 mg/dL
ALT	19	8-70 U/L
Urinalysis	Negative, no casts, RBCs or proteinuria noted	Negative
RPR with TP-PA	Nonreactive	Nonreactive

Her rheumatology workup revealed a high-titer antinuclear antibody (ANA) 1:1280 with a high-titer centromere antibody. antineutrophil cytoplasmic antibodies (ANCA) antibodies were negative (Table 2). Myositis panel was normal. Her clinical examination showed no features associated with scleroderma or myositis.

**Table 2:** Autoimmune Serology Panel Pm/Scl, Polymyositis, scleroderma antibody (Ab); Sm, Smith; RNP, Ribonucleoprotein Ab; SSa/SSb, Sjogren’s ab; RF, Rheumatoid factor; Scl-70, Scleroderma Ab; anti-CCP, Anti-cyclic citrullinated peptide Ab

Test	Observed Value	Reference Range
Antinuclear Antibody	Positive	Negative
ANA Titre	1:1280	<1:80 titre
NDNA (Crithidia) Ab IFA	<1:10	<1:10
Centromere Ab	>8.0	<1.0 U
PM/Scl 100 Ab	Negative	Negative
Sm Ab	<20	<20 U
RNP Ab	<20	<20 U
U3 RNP	Negative	Negative
SSa/SSb	<20/<20	<20/<20 U
RF/ cryocrit	<10/negative	<10 U/negative
Scl-70	0	<20 U
Anti-CCP Ab	<20	<20 U

Following persistent weakness in her legs, a lumbar puncture was performed (Table 3). A comprehensive infectious disease panel was ordered. Polymerase chain reaction (PCR) CSF bacterial, atypical and viral cultures were negative. These included herpes zoster (VZV), herpes simplex (HSV), Cytomegalovirus (CMV), West Nile, Parvovirus, JC virus, BK virus, Epstein-Barr (EBV), and HIV. CSF cytology was negative for malignant cells. Encephalopathy paraneoplastic and autoimmune panels were negative as well. The dengue fever IgM antibody was negative, but IgG was elevated to 2.29 (with 0.80 being negative). The chikungunya IgM and IgG antibodies were negative. 

**Table 3:** Cerebrospinal Fluid (CSF) results RBC, Red Blood cell; WBC, White blood cell; IgG, Immunoglobulin; ACE, Angiotensin Converting Enzyme.

Test	Observed Value	Reference Range
CSF Appearance	Pink and Clear	Colorless
RBC Count	69	0-10/cmm
WBC Count	4	0-5/cmm
Segmented Neutrophils	1	1%
Lymphocyle	89	40-80%
Monocytes	10	15-45%
Plasma Cells	0	0
Glucose, CSF	98	43-73 mg/dL
Protein, CSF	49	15-45 mg/dL
Albumin IgG Synthesis	3083	3500-5200 mg/dL
Albumin Index	9.4	0.0-8.9
IgG Index	0.6	0-0.6
Oligoclonal Bands	Negative	0-1 bands
ACE level	1.5	0-2.5

Electromyography (EMG) studies revealed abnormal findings consistent with sensorimotor polyneuropathy with mixed axonal and demyelinating features, which made a myopathic process less likely. Needle EMG of select muscles of the right lower extremity demonstrated fibrillation potentials and positive sharp waves in the tibialis anterior (TA), medial gastrocnemius and vastus medialis. On activation, the right TA demonstrated normal amplitude, prolonged duration (>12ms) Motor Unit Action Potentials (MUAPs) with reduced recruitment. The medial gastrocnemius demonstrated normal MUAP morphology with reduced recruitment. The vastus medialis demonstrated normal MUAP morphology with normal recruitment. 

Extensive diagnostic imaging was performed, including CT scans of the chest, abdomen, and pelvis, all of which ruled out malignancy. A pelvic ultrasound was also negative for pathology.

**Figure 1:**
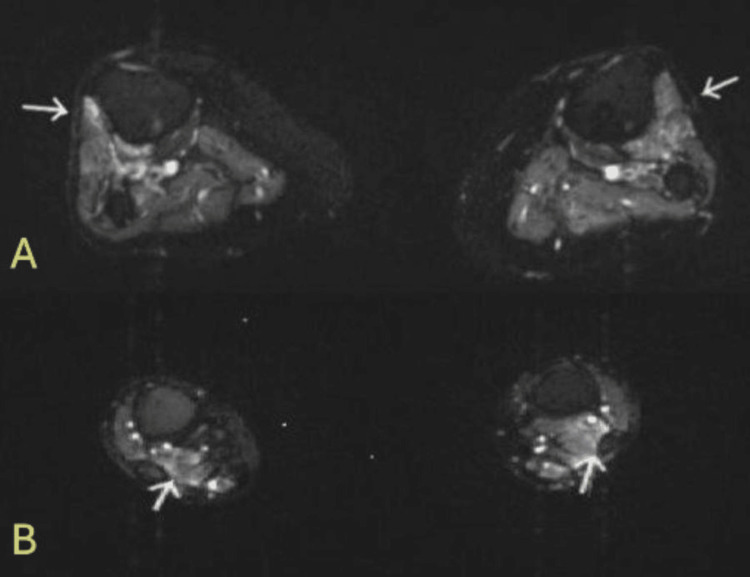
Bilateral MRI tibia and fibula without contrast, T2-weighted axial images A: arrows point to fluid-sensitive enhancement around the anterior tibialis muscles bilaterally. B: arrows point to fluid-sensitive enhancement around the flexor hallucis longus muscles bilaterally.

MRI of the lumbar spine demonstrated degenerative disc disease with mid-neuroforaminal stenosis, this imaging was not available. MRI of the tibia and fibula non-contrast revealed patchy edema around the anterior tibialis (Figure 1A) and flexor hallucis longus muscles (Figure 1B).

**Figure 2:**
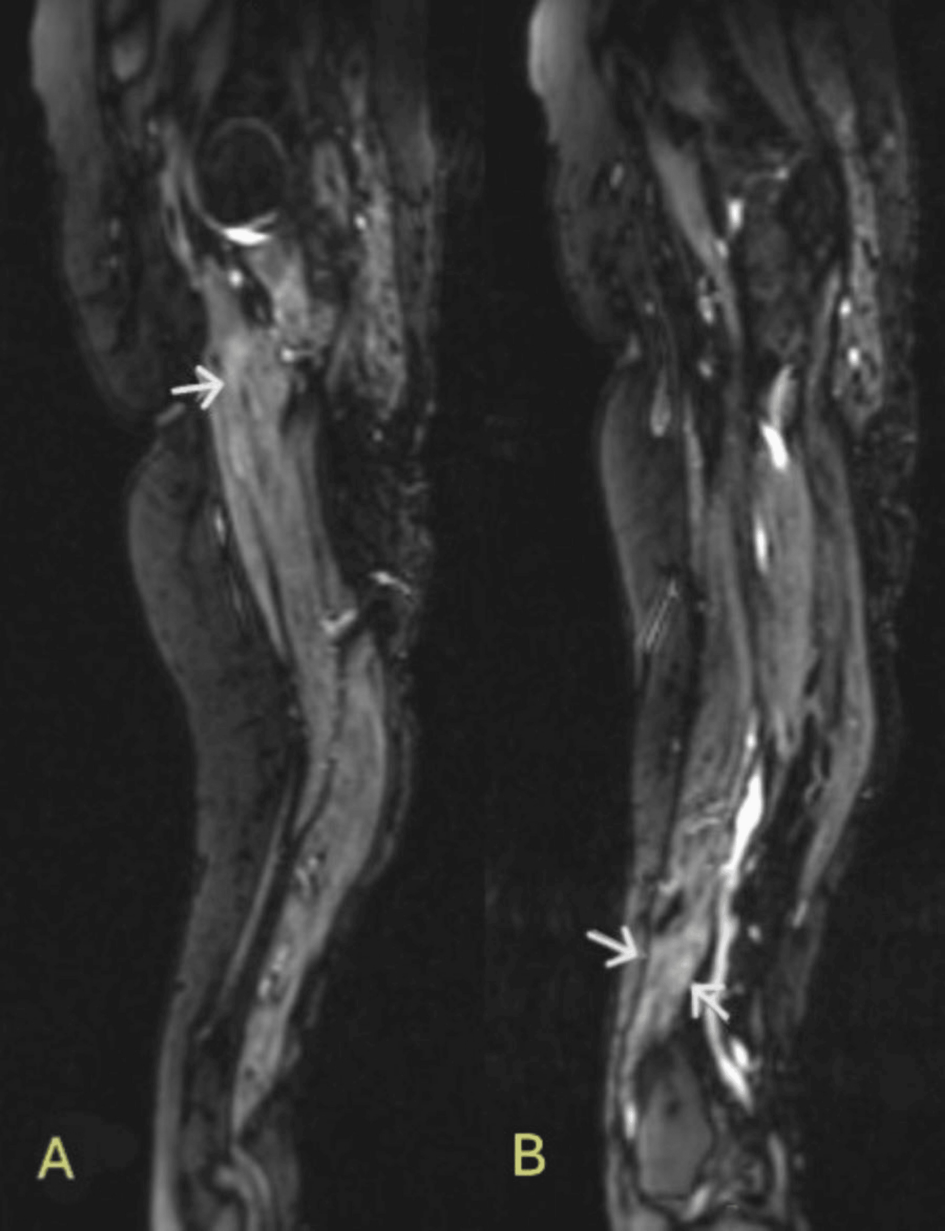
Bilateral MRI femur non contrast, T2 weighted sagittal images A: arrows point to fluid-sensitive enhancement around the adductor muscles. B: arrows point to fluid-sensitive enhancement around the vastus medialis muscles.

MRI of femurs without contrast revealed muscle edema about the origin of the adductor muscle on both sides (Figure 2A). Mild patchy edema was noted about the vastus medialis muscle as well (Figure 2B). These findings were described as neurogenic, with a differential diagnosis of vasculitis, diabetic sequelae or infectious neuritis. Given that the picture did not fit vasculitis, and her diabetes was moderately well controlled (A1c 6.9), the radiology findings were attributed to the underlying viral infection.

A multidisciplinary team discussion was held regarding the potential sural nerve biopsy and use of intravenous immunoglobulin (IVIG). The team felt that the yield of a sural nerve biopsy would be low and the patient had clinically improved at this point. The team concluded that IVIG treatment was unlikely to provide significant benefit at this stage in her clinical course, as her inflammatory process was no longer considered acute and the patient showed significant response to physical therapy. She was subsequently discharged to an acute rehabilitation facility for continued physical and occupational therapy to address her remaining functional limitations.

## Discussion

This case presents compelling features of post-viral sensorimotor polyneuropathy with foot drop. The patient's clinical course suggests a causal relationship between the initial viral infection and subsequent neurological deterioration. The presence of lymphocytic vasculitis on skin biopsy, combined with the EMG findings of mixed sensorimotor polyneuropathy, indicates a possible immune-mediated process triggered by the viral infection.

Dengue and chikungunya infections are mosquito-borne viral diseases that typically present as acute febrile illness. Dengue fever, caused by the Dengue virus (DENV), characteristically manifests with fever, severe headache, retro-orbital pain, severe myalgias and arthralgias ("breakbone fever"), and rash. Severe manifestations can include hemorrhagic fever and shock syndrome. Chikungunya, caused by the Chikungunya virus (CHIKV), presents with sudden-onset high fever, severe polyarthralgia, myalgia, headache, and maculopapular rash. The name "chikungunya" derives from the Kimakonde language, meaning "to become contorted," describing the stooped appearance of sufferers due to severe joint pain [7].

The spectrum of neurological complications associated with both dengue and chikungunya infections is broad and significant. Dengue virus has been documented to cause various neurological manifestations including encephalitis, encephalopathy, and Guillain-Barré syndrome. Additionally, cases of myelitis, myositis, acute disseminated encephalomyelitis, neuromyelitis optica, and peripheral neuropathies have been reported in the literature. Similarly, chikungunya virus can affect both the central and peripheral nervous systems, manifesting as meningoencephalitis, myelopathy, peripheral neuropathy, Guillain-Barré syndrome, acute flaccid paralysis, and neuroretinitis [8].

While encephalitis and encephalomyelitis have been commonly reported, there have been very few cases of mononeuropathies associated with dengue. In a similar case, mononeuritis in the form of cranial nerve palsy and foot drop was observed [9]. The pathogenesis behind dengue or chikungunya-related mononeuropathies is thought to be immune-mediated rather than direct activity of the virus itself. Studies in mice have shown that chikungunya virus infections lead to production of antibodies that function to limit viral replication. It is suspected that such activity results in an immune-mediated post-infectious myelopathy [10].

The concurrent infection with both viruses, as suspected in our patient, may have contributed to the severity and persistence of her neurological symptoms. There have not been many cases of vasculitis associated with dengue or chikungunya infections based on literature review. One case reported central nervous system vasculitis observed in a woman one day after recovery from dengue hemorrhagic fever [11]. Lymphocytic vasculitis can be seen with skin involvement. 

The diagnostic approach to such cases requires careful consideration of the timeline of the illness. In the acute phase, viral detection through non-structural protein 1 (NS1) antigen testing and RT-PCR is crucial for dengue and chikungunya, respectively. As the illness progresses, serological testing becomes more relevant, with IgM antibodies typically appearing by day five of illness. In cases of neurological complications, as demonstrated in our patient, additional testing including EMG/NCV studies, neuroimaging, CSF analysis, and autoimmune panels may be necessary to evaluate the extent of involvement and rule out other etiologies. The positive ANA and centromere antibody findings in our patient suggest an underlying autoimmune predisposition, which may contribute to the development of post-viral complications [12-14].

Treatment of these neurological complications requires a multi-faceted approach. The acute viral infection typically requires supportive care, including careful fluid management and pain control. When neurological complications develop, targeted interventions may be necessary. These can include immunotherapy for post-infectious inflammatory conditions, IVIG or plasmapheresis for Guillain-Barré syndrome, and corticosteroids for inflammatory neuropathies. Physical therapy and rehabilitation play crucial roles in functional recovery, particularly in cases of focal palsies such as foot drop, as seen in our patient. The use of orthotics or splinting may be necessary to improve mobility and prevent further complications [15].

Prevention remains the cornerstone of management for these mosquito-borne illnesses. Effective prevention requires a comprehensive approach incorporating vector control measures, personal protection strategies, and community-level interventions. Vector control includes elimination of mosquito breeding sites and appropriate use of insecticides. Personal protection using mosquito repellents and appropriate clothing is essential, particularly in endemic areas. Community-level interventions, including public health education and environmental management, play vital roles in reducing disease transmission. The effects of climate change on the cascading risks of infectious diseases are well documented. Global warming has contributed to a climate more suitable for the mosquitoes carrying dengue, chikungunya, and other infectious organisms. This can result in longer transmission seasons and facilitate increases in geographic range. Diseases once isolated in tropical climates could expand into temperate zones [16].

The prognosis of neurological complications varies significantly among patients and depends on several factors. Early recognition and intervention lead to better outcomes, while delayed diagnosis may result in prolonged recovery or permanent deficits. Age and comorbidities play important roles in recovery and are significant risk factors, with younger patients typically showing better outcomes [17]. The type of neurological involvement also impacts prognosis, with isolated peripheral nerve involvement having a more favorable course compared to central nervous system involvement. Infections are known mimickers of vasculitis with the ability to affect any vessel size [18]. Depending on clinical history, dengue or chikungunya should be considered as differential diagnoses when neurological deficits such as mononeuritis manifest.

The serological evidence of past dengue infection in our patient, coupled with the initial clinical diagnosis of concurrent chikungunya infection, raises important questions about the potential synergistic effects of these viruses on neurological outcomes. While direct causation is difficult to establish, the temporal relationship and exclusion of other common causes support the association. The presence of autoimmune markers suggests a mechanism for post-infectious neurological complications, although the negative results for other autoimmune markers argue against primary autoimmune disease as the main cause of the patient's symptoms.

## Conclusions

This case highlights the potential for severe neurological complications following tropical viral infections, particularly in elderly patients with underlying medical conditions. The persistent foot drop and polyneuropathy demonstrate the importance of close monitoring for neurological sequelae in patients with dengue and chikungunya infections. Further research is needed to better understand the mechanisms of post-viral neurological complications and to develop targeted therapeutic approaches.
